# Effects of Octylphenol and Bisphenol A on the Metal Cation Transporter Channels of Mouse Placentas

**DOI:** 10.3390/ijerph13100965

**Published:** 2016-09-28

**Authors:** Jae-Hwan Lee, Changhwan Ahn, Hee Young Kang, Eui-Ju Hong, Sang-Hwan Hyun, Kyung-Chul Choi, Eui-Bae Jeung

**Affiliations:** 1 Laboratory of Veterinary Biochemistry and Molecular Biology, Veterinary Medical Center and College of Veterinary Medicine, Cheongju, Chungbuk 28644, Korea; phantom4015@nate.com (J.-H.L.); prac@naver.com (C.A.); nannaingir@hanmail.net (H.Y.K.); 2 Laboratory of Veterinary Biochemistry, College of Veterinary Medicine, Chungnam National University, Daejeon 34134, Korea; ejhong@cnu.ac.kr; 3 Laboratory of Veterinary Biotechnology and Embryology, College of Veterinary Medicine, Chungbuk National University, Cheongju, Chungbuk 28644, Korea; shhyun@cbu.ac.kr; 4 Laboratory of Biochemistry and Immunology, College of Veterinary Medicine, Chungbuk National University, Cheongju, Chungbuk 28644, Korea; kchoi@cbu.ac.kr

**Keywords:** endocrine-disrupting chemicals, octylphenol, bisphenol A, cation transport

## Abstract

Octylphenol (OP) and bisphenol A (BPA) are known as endocrine-disrupting chemicals (EDCs). During pregnancy, the expression of steroid hormone receptors is controlled by maternal and fetal nutrition. To evaluate the impact of EDCs during pregnancy, ethinyl estradiol (EE, 0.2 mg/kg/day), OP (50 mg/kg/day), and BPA (50 mg/kg/day) were administered to pregnant mice. The mRNA levels of TRPV6 (transient receptor potential cation channels in subfamily V, member 6) decreased significantly by EE and OP. The PMCA1 (ATPase, Ca^++^ transporting, plasma membrane 1) mRNA and protein levels decreased significantly by EE, OP, and BPA. CTR1 (solute carrier family 31, member 1) and ATP7A (ATPase, Cu^++^ transporting, alpha polypeptide) expression decreased significantly by EE, OP, and BPA. The mRNA levels of IREG1 (iron-regulated transporter, member 1) decreased significantly by EE. Hephaestin (HEPH) mRNA levels decreased significantly by EE, OP, and BPA, and protein levels decreased significantly by BPA. As a result of immunohistochemistry analysis, all cation transporter proteins were found in labyrinth of placenta. To confirm the cytosolic level of cations, levels of cation level in fetal serum were measured. EE, OP, and BPA significantly reduced serum calcium and copper levels, and iron levels were reduced by BPA. Taken together, some EDCs, such as OP and BPA, could modulate the calcium, copper, and iron ion-transporting channels during pregnancy. The fetus relies on the mother for ionic transportation, and, therefore, pregnant women should avoid exposure to cation-channel-disrupting chemicals.

## 1. Introduction

The placenta is an organ that connects the developing fetus to the uterine wall to allow nutrient uptake, waste elimination, and gas exchange via the mother’s blood supply; prevents internal infection; and produces hormones to support pregnancy. Oxygen, carbon dioxide, cations, and glucose are essential factors in fetal growth. Oxygen, carbon dioxide, and cations are transferred via specific receptors located on cell membranes or cytoplasm in the placenta. Among them, the cation (e.g., calcium, copper, iron, etc.) transfer genes are regulated by estrogen, vitamin D, and human placental lactogen [1,2,3,4].

Endocrine-disrupting chemicals (EDCs) can mimic endogenous hormones, binding hormone receptors and interfering with normal hormonal function, including creating completely different responses from those of the original hormone. EDCs cause a variety of side effects including cancers and reproductive abnormalities. EDCs are found in plastic, pesticides, insecticide, paint, and some baby feeding bottles [5]. Octylphenol (OP) is an alkyl phenol that is mainly used in plastics, pesticides, and insecticides. Bisphenol A (BPA) is an organic compound used for cans and plastic containers and some baby feeding bottles. According to previous research, high blood BPA in the mother increases the risk of miscarriage [6], and EDC exposure during pregnancy may have a significant effect on increasing the risk of developing type 2 diabetes [7]. Exposure to low-dose BPA during gestation and lactation may result in neuronal and glial developmental alterations [8]. BPA and OP have negative effects on the expression of the genes involved in calcium transfer [9]. These chemicals are considered to be EDCs owing to their mimicking the action of endogenous estrogen.

If the fetus cannot receive a proper supply of calcium, copper, and iron, many diseases can occur. Calcium deficiency causes hypocalcemia and affects nervous system, musculature, and the heart. Excess calcium causes kidney stones, fatigue, and hypotension [10]. Developmental diseases of the fetal brain, hair, and bones as well as the growth of blood vessels can occur with a copper deficiency, and excess copper appears as nephritis, vascular rupture, lowered immunity, thyroid dysfunction, and adrenal dysfunction. Iron-deficiency anemia occurs with iron deficiency [11,12]. As such, problems with cation transport during pregnancy can affect fetal growth.

The transient receptor potential cation channels in subfamily V, member 6 (TRPV6) are the calcium entry channels responsible for calcium absorption in the placenta. ATPase, Ca^++^ transporting, plasma membrane 1 (PMCA1) excretes calcium into the fetus [13]. Solute carrier family 31, member 1 (CTR1) is expressed early in pregnancy, and copper enters into the placenta through CTR1. ATPase, Cu^++^ transporting, alpha polypeptide (ATP7A) is involved in maintaining the homeostasis of copper, and it serves to provide the copper to the fetal blood [13]. Iron is transported to the fetus through the solute carrier family 40 (iron-regulated transporter), member 1 (IREG1). The iron is oxidized to Fe^3+^ by a protein called hephaestin (HEPH), a copper oxidase that binds to serum transferrin [14].

EDCs are known to affect either the fetus or the mother during pregnancy, according to previous studies. Estrogen induces changes in channel protein expression, and EDCs are known to mimic estrogen [15,16,17,18]. In this study, OP and BPA were hypothesized to induce changes in channel protein expression. To verify this hypothesis, we administered OP and BPA to pregnant mice and examined the cation transport gene expression in the placentas, determined localization of the cation transport genes, and measured the cation levels in the fetal serum.

## 2. Materials and Methods

### 2.1. Chemicals

EE (ethinyl estradiol), OP (octylphenol) and BPA (bisphenol A) were purchased from Sigma-Aldrich Corp. (St. Louis, MO, USA). Stock solutions were made by dissolving in DMSO (dimethyl sulfoxide; Santa Cruz Biotechnology, Santa Cruz, CA, USA) and diluted with corn oil (Sigma-Aldrich) when needed.

### 2.2. Animals

Female ICR mice (8 weeks old; weighing 25–30 g) were obtained from Koatech (Pyeongtaek, Gyeonggi, Korea). All mice were housed in polycarbonate cages and acclimated in an environmentally controlled room. Temperature of the environment was set at 23 ± 2 °C with 50% ± 10% relative humidity and a 12 h light–dark cycle. Female mice were mated with adult male ICR mice (10 weeks old) overnight and then examined for the presence of vaginal plug the following morning. The day that vaginal plugs were observed was set as GD (gestational date) 0.5. From GD 11.5 to GD 16.5, the pregnant mice were divided into four groups of five animals each and given subcutaneously administered EE (0.2 mg/kg/day; positive control), OP (50 mg/kg/day), and BPA (50 mg/kg/day) all dissolved in corn oil (Sigma-Aldrich). Mice were sacrificed on GD 17.5. All animal procedures were approved by the Ethics Committee of Chungbuk National University (CBNUA-791-15-01).

### 2.3. Total RNA Extraction and Quantitative Real-Time PCR

Placenta were washed with cold, sterile saline and homogenized in TRIzol with a bullet blender (Next Advance, Averill Park, NY, USA). Total RNA was extracted from the solution using TRI reagent (Ambion, Austin, TX, USA) according to the manufacturer’s protocol. Total RNA concentration was measured by using an Epoch Microplate Spectrophotometer. RNA (1 μg) was transcribed using mMLV (Moloney murine leukemia virus) reverse transcriptase (iNtRON Bio, Gyeonggi-do, Korea) with random 9-mer primer (TaKaRa Bio Inc., Shiga, Japan) to produce first-strand complementary DNA (cDNA). The cDNA template (1 μL) was assayed using SYBR PCR (TaKaRa Bio Inc.) real-time PCR according to the manufacturer’s protocol. Real-time PCR was performed under the following conditions: 40 cycles of denaturation at 95 °C for 30 s, annealing at 58 °C for 30 s, and extension at 72 °C for 30 s. Fluorescent intensity was measured at the end of the extension phase of each cycle. The threshold value for fluorescence intensity for all samples was set manually. The PCR cycle at which the fluorescence intensity threshold was in the exponential phase of PCR amplification was designated as the CT (threshold cycle). The CT value was determined automatically at the exponential phase of the delta CT fluorescence detection graph. The PCR product of *Rn18S (18S ribosomal RNA)* was used as an internal control for normalization. Primer sequences for the *Trpv6*, *Pmca1*, *Ctr1*, *Atp7a*, *Ireg1*, *Heph*, and *Rn18S* are shown in Table 1. The amount of transcript present was inversely related to the observed CT, and CT was expected to increase by 1 for every 2-fold dilution in the amount of transcript. Relative expression was calculated using the equation R = 2 ^– (ΔCTsample – ΔCTcontrol]^. To determine a normalized arbitrary value for each gene, every data point was normalized to the control gene, as well as to their respective controls.

### 2.4. Western Blotting

Placenta were washed with cold sterile saline and homogenized in Pro-prep (iNtRON) with a bullet blender (Next Advance). The homogenate or lysate was cleared by centrifugation at 13,000 RPM for 10 min at 4 °C. Next, 30 μg of sample were treated by mixing with sodium dodecylsulfate (SDS) sample buffer, heating at 60 °C for 10 min, and centrifuging 13,000 RPM at 4 °C for 10 min. Afterwards, 10% SDS and 30% acrylamide was prepared and electrophoresis was performed. The separated proteins were transferred to PVDF (polyvinylidene fluoride) membrane (Merck Millipore, Taunton, MA, USA). The membrane was blocked with 5% skim milk dissolved in TBS-T for 1 h. The membrane was then incubated with primary antibodies for 3 h at RT or overnight at 4 °C: anti-TRPV6 (Santa Cruz Biotechnology), anti-PMCA1 (Swant), anti-CTR1 (Novusbio), anti-ATP7A (Santa Cruz Biotechnology), anti-IREG1 (Abcam), anti-HEPH (Abcam), and anti-α-tubulin (Cell Signaling Technology) diluted 1000-fold in 1% BSA (bovine serum albumin). Next, the membrane was washed four times for 10 min each with TBS-T. The blot was subsequently incubated for 1 h with secondary antibody conjugated with HRP (horseradish peroxidase) (rabbit polyclonal, mouse polyclonal, goat polyclonal; Santa Cruz Biotechnology) diluted 3000-fold in 2.5% non-fat milk dissolved in TBS-T. The membrane was washed four times for 10 min each with TBS-T. ECL (enhanced chemiluminescence) reagent (Santa Cruz Biotechnology) with a CCD (charge-coupled device) was used to detect antibody binding. Using the Chemi Doc equipment, GenGnome 5 (Syngene, Cambridge, UK). The optical density of the target band was analyzed by image J (NIH, Bethesda, MD, USA).

### 2.5. Immunohistochemical Analysis

Localization of TRPV6, PMCA1, CTR1, ATP7A, IREG1, and HEPH proteins was examined by immunohistochemistry. Mouse placenta tissues were embedded in paraffin, cut into sections (4 μm thick), deparaffinized in xylene, and hydrated in descending grades of ethanol. Endogenous peroxidase activity was blocked with 3% hydrogen peroxide in TBS-T for 30 min. Nonspecific reactions were blocked by incubating the sections in 10% normal goat serum (Vector Laboratories, Burlingame, CA, USA) for 1 h at room temperature. The sections were subsequently incubated overnight at room temperature with antibodies against TRPV6 (1:300, Santa Cruz Biotechnology), PMCA1 (1:300, Swant), CTR1 (1:300, Novusbio), ATP7A (1:300, Santa Cruz Biotechnology), IREG1 (1:300, Abcam), or HEPH (1:300, Abcam) diluted in 1% BSA. After washing with TBS-T, the sections were incubated with a biotinylated secondary antibody (rabbit or mouse IgG, Vector Laboratories) for 1 h at 37 °C and then incubated with ABC Elite (Vector Laboratories) for 30 min at 37 °C. Diaminobenzidine (DAB; Sigma-Aldrich) was used as a chromogen. The sections were counterstained with hematoxylin and mounted in Cytoseal* 60 Mounting Medium (Richard-Allan Scientific Co., Kalamazoo, MI, USA).

### 2.6. Fetal Serum Collection and Measurement of Blood Calcium, Copper, and Iron Concentration

Blood was collected from each mouse fetus, transferred to serum separator tubes (BD Caribe, Ltd., Franklin Lakes, NJ, USA) and centrifuged at 400× *g* for 15 min. Serum calcium, copper, and iron concentrations were measured using an inductively coupled plasma optical emission spectrometer (ICP-OES).

### 2.7. Statistical Analysis

The results of all experiments are presented as the mean ± SD. Data were analyzed with a nonparametric one-way analysis of variance (ANOVA), using the Tukey’s test for multiple comparisons. Statistical analysis was performed using Prism Graph Pad software (v5.0; GraphPad Software Inc., La Jolla, CA, USA). *p*-values < 0.05 was considered statistically significant.

## 3. Results

### 3.1. Expression of Calcium Transporter Channels in Response to Octylphenol and Bisphenol A

**Figure 1:**
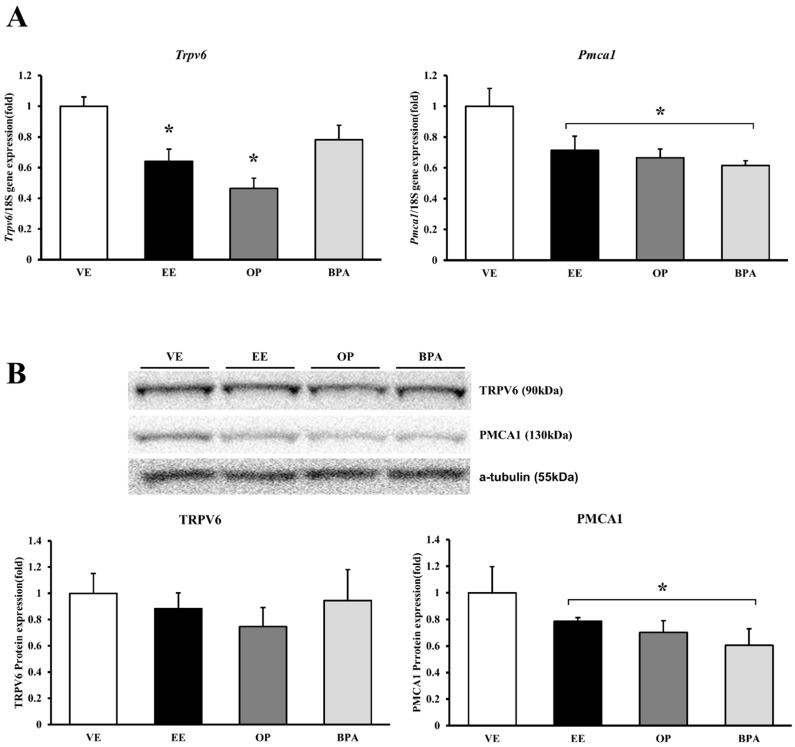
mRNA and protein expression of calcium transporter channel in mouse placenta. The expression of calcium-transporting channel gene was examined by real-time PCR, and normalized by *Rn18S*. Protein expression was examined by Western blotting. mRNA expression of *Trpv6* and *Pmca1* (**A**); protein expression of TRPV6 and PMCA1 (**B**). * *p* < 0.05 vs. VE. Data are presented as the mean ± SD. VE DMSO 5%, ethinyl estradiol (EE) 0.2 mg/kg, octylphenol (OP), bisphenol A (BPA) 50 mg/kg.

The transcript and calcium-transporting channel protein expression determined the biological pathway involved in cation ions following the BPA and OP administration. The *Trpv6* levels decreased significantly by EE (64% vs. vehicle, *p* < 0.05) and OP (46% vs. vehicle, *p* < 0.05) compared with the vehicle control. Although TRPV6 expression protein levels did not change significantly, they did decrease. Interestingly, the *Pmca1* mRNA levels decreased significantly by EE (29% vs. vehicle, *p* < 0.05), OP (33% vs. vehicle, *p* < 0.05), and BPA (38% vs. vehicle, *p* < 0.05) compared with the vehicle control. Similarly, PMCA1 protein expression patterns were consistent with those of mRNA. PMCA1 protein levels decreased significantly by EE (22% vs. vehicle, *p* < 0.05), OP (30% vs. vehicle, *p* < 0.05) and BPA (39% vs. vehicle, *p* < 0.05) administration. The calcium-transporting channel gene expression was confirmed as resulting from reduced EDCs (Figure 1). This result indicates that OP and BPA can lead to disturbances in calcium homeostasis by modulating calcium transporting channel.

### 3.2. Expression of Copper Transporter Channels in Response to Octylphenol and Bisphenol A

**Figure 2:**
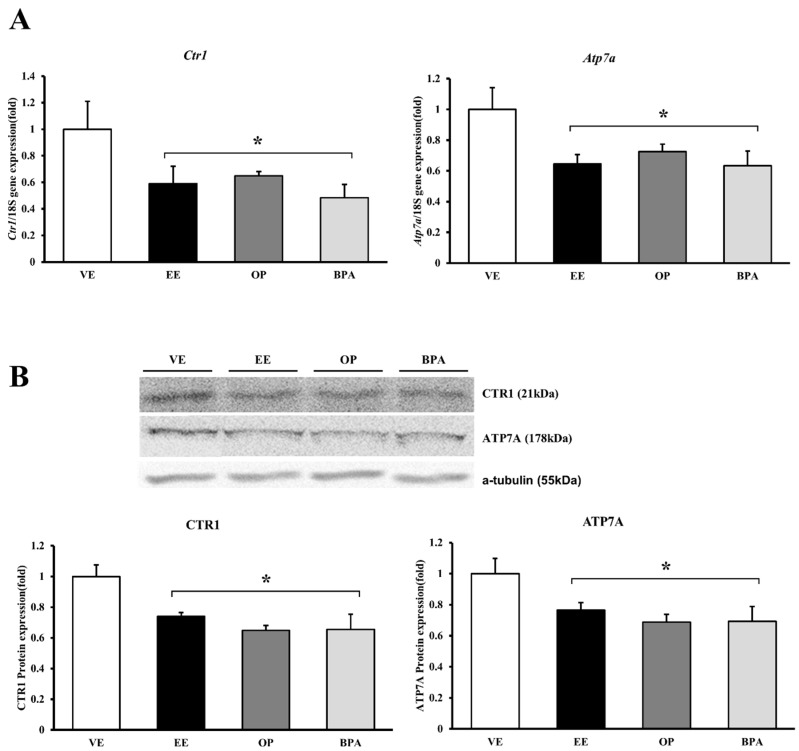
mRNA and protein expression of copper transporter channel in mouse placenta. The expression of calcium-transporting channel gene was examined by real-time PCR, and normalized by *Rn18S*. Protein expression was examined by Western blotting. mRNA expression of *Ctr1* and *Atp7a* (**A**); protein expression of CTR1 and ATP7A (**B**). * *p* < 0.05 vs. VE. Data are presented as the mean ± SD. VE DMSO 5%, EE 0.2 mg/kg, OP, BPA 50 mg/kg.

The transcripts of copper transporter channels were examined by real-time PCR and protein levels were detected by Western blot. *Ctr1* mRNA expression decreased significantly by EE (41% vs. vehicle, *p* < 0.05), OP (35% vs. vehicle, *p* < 0.05), and BPA (51% vs. vehicle, *p* < 0.05). The CTR1 protein expression patterns after administration of EE (26% vs. vehicle, *p* < 0.05), OP (35% vs. vehicle, *p* < 0.05), and BPA (34% vs. vehicle, *p* < 0.05) were similar to those for mRNA expression. *Atp7a* mRNA expression decreased significantly by EE (36% vs. vehicle, *p* < 0.05), OP (28% vs. vehicle, *p* < 0.05), and BPA (37% vs. vehicle, *p* < 0.05). The ATP7A protein expression patterns were the same as those for the transcription levels, and the ATP7A levels decreased by EE (24% vs. vehicle, *p* < 0.05), OP (31% vs. vehicle, *p* < 0.05), and BPA (31% vs. vehicle, *p* < 0.05) (Figure 2). OP and BPA can affect copper transport channel gene expression in maternal–fetal copper homeostasis.

### 3.3. Expression of Iron Transporter Channels in Response to Octylphenol and Bisphenol A

**Figure 3:**
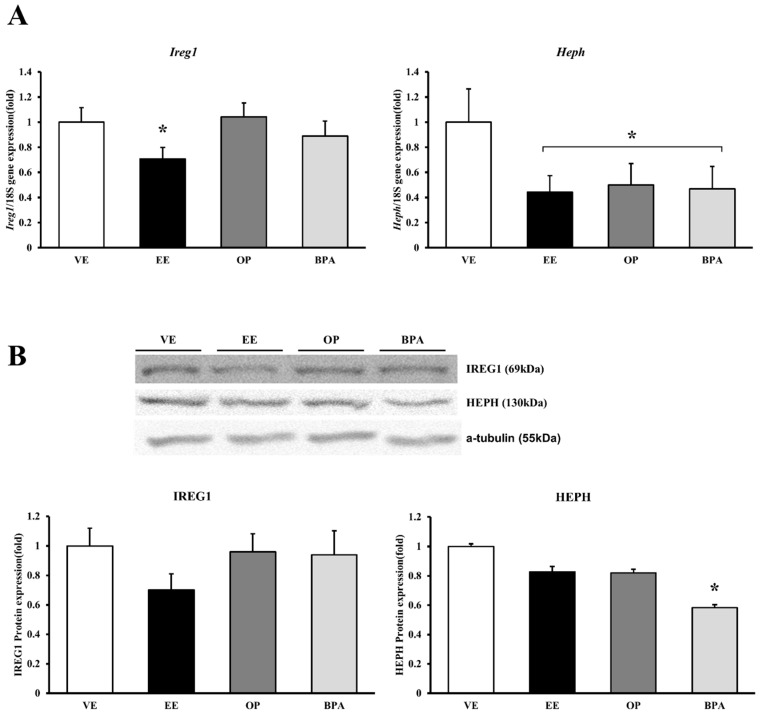
mRNA and protein expression of iron transporter channel in mouse placenta. The expression of calcium-transporting channel gene was examined by real-time PCR, and normalized by *Rn18S*. Protein expression was examined by Western blotting. mRNA expression of *Ireg1* and *Heph* (**A**); protein expression of IREG1 and HEPH (**B**). * *p* < 0.05 vs. VE. Data are presented as the mean ± SD. VE DMSO 5%, EE 0.2 mg/kg, OP, BPA 50 mg/kg.

mRNA and protein expression levels were measured using real-time PCR and Western blot, respectively. The *Ireg1* mRNA expression decreased significantly by EE (29% vs. vehicle, *p* < 0.05). IREG1 protein expression decreased by EE, but it did not change significantly. *Heph* mRNA expression decreased significantly by EE (56% vs. vehicle, *p* < 0.05), OP (50% vs. vehicle, *p* < 0.05), and BPA (53% vs. vehicle, *p* < 0.05), but HEPH protein expression only decreased by BPA (42% vs. vehicle, *p* < 0.05) (Figure 3). Iron transport channel problems can lead to homeostatic disturbances.

### 3.4. Expression Localization of Cation Transporter Channels in Mouse Placenta

**Figure 4:**
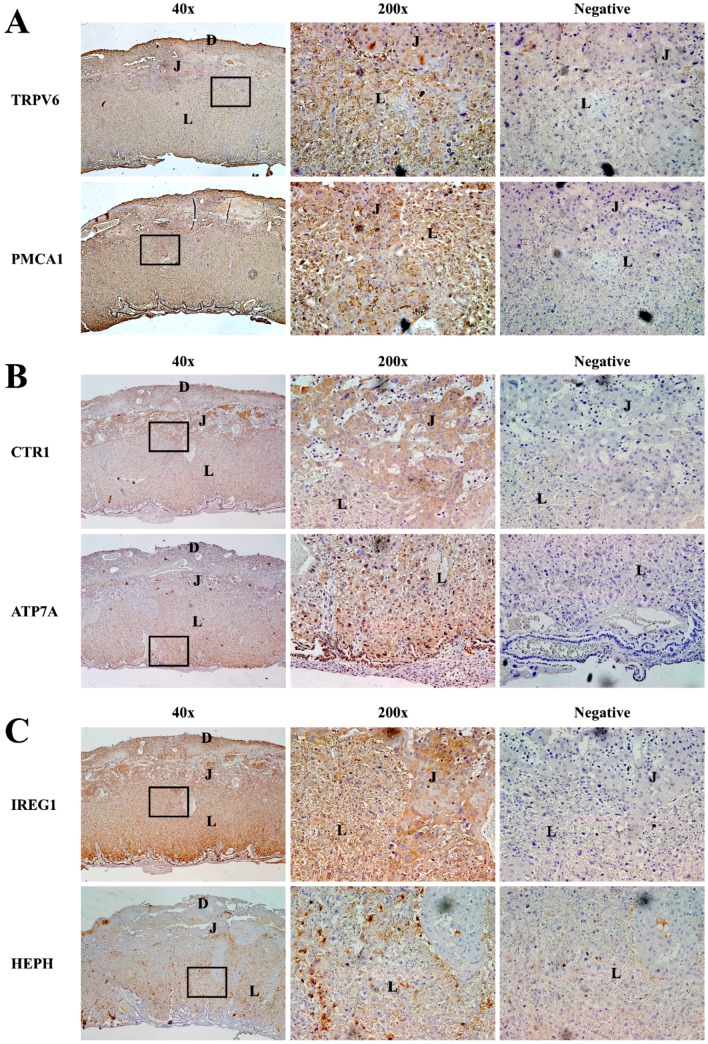
Localization of cation transporter channels in mouse placenta. Localization of calcium (**A**), copper (**B**), and iron (**C**) transporter channel in the placenta of pregnant mice were confirmed using immunohistochemistry. Cation transporter channel proteins on gestational date (GD) 17.5. (**Left**): 40× magnified view of placenta, (**Mid**): 200× magnified placenta region containing labyrinth (L), junctional (J), decidua (D) and (**Right**): 200× negative controls were stained in the absence of primary antibodies. Regions containing brown color indicate cation transporter channel expression.

Because administering EE, OP, and BPA altered the cation-transporting channel expression, we examined expression localization of the channels in the placenta; we monitored this localization at GD 17.5. Immunohistochemical analysis was used to determine TRPV6, PMCA1, CTR1, ATP7A, IREG1, and HEPH localization using specific antibodies (Figure 4) [19]. The placental tissues were composed of three representative layer structures: labyrinth, junctional, and decidua [13], and TRPV6 and PMCA1 proteins were found in all three layers (Figure 4A). Although CTR1 protein was found in all three layers of placenta, their locations were particularly confirmed in many junctional zones. ATP7A protein was found in labyrinth and decidua of placenta, and IREG1 protein was found in all three layers of placenta (Figure 4B). HEPH protein was mainly detected in the labyrinth (Figure 4C). All cation transporter channels were found in labyrinth of placenta because the labyrinth zone consists of fetal and maternal blood spaces separated by vascular endothelium and specialized trophoblast cell types. The labyrinth zone provides the interface for exchanges between maternal and fetal circulation [20].

### 3.5. Octylphenol and Bisphenol A Influence on Fetal Cations

**Figure 5:**
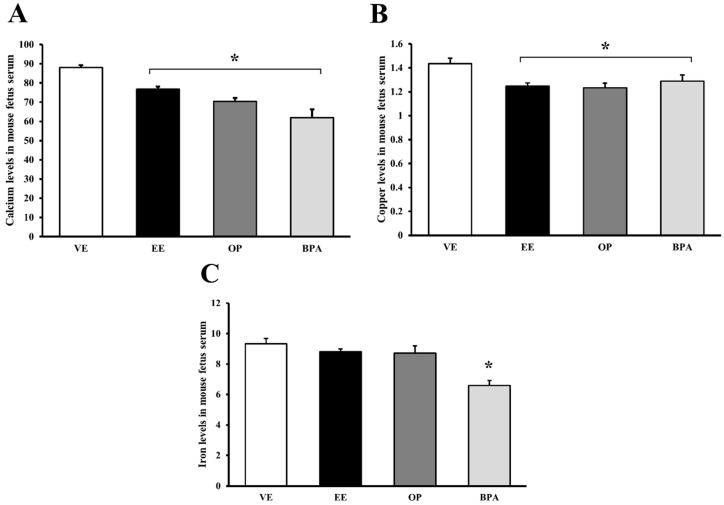
Alteration of cation levels in mouse fetal serum. The effects of OP and BPA were evaluated on placenta cation transporter channel during pregnancy. Calcium (**A**); copper (**B**); and iron (**C**) levels were measured using an inductively coupled plasma optical emission spectrometer (ICP-OES) in fetal serum. * *p* < 0.05 vs. VE. Data are presented as the mean ± SD. VE DMSO 5%, EE 0.2 mg/kg, OP, BPA 50 mg/kg.

The fetal blood was collected fetal blood on GD 17.5 and measured the fetal serum cation levels (Figure 5). Calcium decreased significantly by EE (13% vs. vehicle, *p* < 0.05), OP (20% vs. vehicle, *p* < 0.05), and BPA (30% vs. vehicle, *p* < 0.05) (Figure 5A). Copper decreased significantly by EE (13% vs. vehicle, *p* < 0.05), OP (14% vs. vehicle, *p* < 0.05), and BPA (10% vs. vehicle, *p* < 0.05) (Figure 5B). Iron decreased significantly by BPA (29% vs. vehicle, *p* < 0.05) (Figure 5C). The OP and BPA were confirmed to decrease the cation transport to the fetus.

## 4. Discussion

During pregnancy, calcium, copper, and iron are important cations for fetal growth and development. The fetus requires considerable amounts of these elements to grow. In a previous study, EE, OP, and BPA regulated the expression of calbindin-D_9k_ (CaBP-9k), TRPV6, and TRPV5 in the intestine and kidney during pregnancy in mice [9]. Calcium as well as copper and iron transport channels are regulated by steroid hormones or EDCs such as alkylphenol [21,22].

TRPV6 expression of the calcium-transporting channel decreased by EE and OP but did not change significantly. Another calcium-transporting channel, PMCA1, was downregulated by EE, OP, and BPA, which might have influenced the reduced fetal calcium level. In the present study, we also confirmed reduced calcium ions in fetal serum following synthetic estrogen or EDC exposure in pregnant animals. EE, OP, and BPA reduced the concentrations of serum calcium in pregnant mice in a previous study [9]. This is consistent with the reduction of placental TRPV6 and PMCA1 expression that would be expected for low levels of calcium in fetal serum. As with the calcium-transporting channels, copper-transporting channel expression decreased with EE, OP, and BPA; both CTR1 and ATP7A expression are also linked with decreased fetal copper. A previous study confirmed that the pathophysiological mechanisms of estrogen intake are linked with copper accumulation in various tissues [23]. For example, CTR1 locates within the cytoplasm and at the lateral membrane of the cells by estrogen [24]. Furthermore, estrogen stimulates copper transport by increasing ATP7A expression and reducing ATP7B expression [25]. When ATP7A protein delivers copper to the fetus via the placenta, excess copper in the fetal blood returns to the maternal blood by ATP7B protein [26]. Both CTR1 and ATP7A expression decreased with synthetic estrogen and with the OP and BPA, and the latter supports the low levels of copper in the fetal serum. As an iron-transporting channel, HEPH in the BPA group decreased by approximately 42% compared with the vehicle control. Recent data have suggested that the basolateral transfer of iron absorption is likely to play a major role in how the intestine responds to changes in systemic iron demands, and both IREG1 and HEPH are the most important factors in regulating iron during pregnancy. These proteins are regulated by endogenous estrogen [27]. In addition, when estrogen was injected into ovariectomized rats at concentrations analogous to those found in late pregnancy, iron absorption increased [28,29]. As estrogen-responsive genes, IREG1 and HEPH are associated with iron and heme metabolism in the mouse uterus. In particular, IREG1 increased in uterine epithelial cells to alter the flux of intra- and intercellular iron levels in response to steroid hormones such as estrogen. These finding indicate that endogenous estrogen has important role in iron metabolism via HEPH and IREG1 regulation [30]. Although fetal serum iron levels decreased in the BPA-treated groups, both IREG1 and HEPH expression might be not involved with iron transport in the placenta because both iron channels decreased in the EE- and octylphenol-treated groups without reduced fetal iron.

Hormones such as progesterone and estrogen require high concentrations in order to maintain a pregnancy [31,32]; progesterone levels are especially high during pregnancy. By exposing various pregnancy-maintaining hormones such as estrogen, EDCs could show weak effects [33,34].

The purpose of this study was to identify the effects and mechanisms of maternal exposure to OP and BPA in the maternal–fetal ionic exchange. Trophoblasts, a type of placental cells, play a major role in implantation and formation of the maternal–fetal interface [35]. It has been reported that the mouse labyrinth and human trophoblasts are present at maternal–fetal interface regions [36]. The concentration of OP and BPA was set up by a previous experiment data on pregnant mice [9,37,38,39]. Given that (1) pregnancies were under unique circumstances and (2) the short gestation period of mice, our treatments were set in high-dose short-term. After treatments with OP and BPA, reduction in the cation transport channel was confirmed. When treated with the OP and BPA, as identified in the results, fluctuations in cation transport channel were observed. These dysregulated cation transport channel could disrupt maternal–fetal ionic homeostasis. According to previous studies, calcium deficiency causes hypocalcemia and imbalances in the bones and brain cell growth. Due to deficiency of copper, bone and vascular formation does not normally occur. Iron deficiency causes iron-deficiency anemia and decreased brain iron [10,11,38,40]. By administering EE, we confirmed alteration of estrogen-responsive cation-transporting channel expression. With the BPA and OP administration, we observed fewer differences in mRNA and protein expression compared with the effects of EDCs in nonpregnant animals [41]. When placental tissues are composed of three representative layer structures, the labyrinth zone provides the interface for exchange between maternal and fetal circulation [42,43]. Many cation transporter channels have been found in the labyrinth of the placenta. Based on comparing cation concentrations in fetal serum, we found that OP and BPA disrupted cation transport via the maternal–fetal placenta. The lower cation levels in fetal serum after administration of EDCs might result from the reduced cation levels in maternal blood [9]. However, since cation transport genes are reduced in the placenta, it can be expected that this could reduce the level of cations in the fetus.

## 5. Conclusions

In conclusion, our results demonstrated that EE, OP, and BPA regulated the expression of cation transporter channels in the placenta during pregnancy. Cation transport from mother to fetus was disrupted with EE, OP, and BPA and estrogenic compounds. Therefore, as expected, the cations in serum of fetus have been reduced. As written in the introduction, if the fetus cannot receive a proper supply of cations, many diseases and negative effects on fetal growth can occur. According to our results, EE, OP, and BPA could have negative effects on fetal growth by interfering with cation transport from mother to fetus.

## Figures and Tables

**Table 1:** Oligonucleotide sequences for quantitative real-time PCR.

Species	Gene	Primer Sequences (5′→3′)	Accession Number
Mouse	*Trpv6*	F: GCTGGTTCTTGAGGGTGGAA	NM_022413.4
		R: ATAGGCACCAAAGGGACGTG	
	*Pmca1*	F: GCACCAAGTTGAAAACATCTCCC	NM_026482.2
		R: TCTCCACAAAGTGCATTATCCCC	
	*Ctr1*	F: TATGAACCACACGGACGACAA	NM_175090.4
		R: GCCATTTCTCCAGGTGTATTGA	
	*Atp7a*	F: TGGGAAAGTGAATGGTGTCCA	NM_001109757.2
		R: ACGGTATTGGTTAAGACAGGGA	
	*Ireg1*	F: GGGTGGATAAGAATGCCAGAC	NM_016917.2
		R: CCTTTGGATTGTGATCGCAGT	
	*Heph*	F: GCAGTGGAACTATGCTCCCAA	NM_001159627.1
		R: CAGCCTGTAACAGTGGTCCTA	
	*Rn18S*	F: CTCAACACGGGAAACCTCAC	NC_000072
		R: CGCTCCACCAACTAAGAACG	

F, forward; R, reverse.
